# Alcohol consumption and physical functioning among middle-aged and older adults in Central and Eastern Europe: Results from the HAPIEE study

**DOI:** 10.1093/ageing/afu083

**Published:** 2014-06-30

**Authors:** Yaoyue Hu, Hynek Pikhart, Sofia Malyutina, Andrzej Pajak, Ruzena Kubinova, Yuri Nikitin, Anne Peasey, Michael Marmot, Martin Bobak

**Affiliations:** 1 Department of Epidemiology and Public Health, University College London, London, UK; 2 Institute of Internal and Preventive Medicine, Siberian Branch of the Russian Academy of Medical Sciences, Novosibirsk, Russian Federation; 3 Novosibirsk State Medical University, Novosibirsk, Russian Federation; 4 Collegium Medicum, Jagiellonian University, Krakow, Poland; 5 National Institute of Public Health, Prague, Czech Republic

**Keywords:** ageing, alcohol consumption, Central and Eastern Europe, older people, physical functioning

## Abstract

**Background:** light-to-moderate drinking is apparently associated with a decreased risk of physical limitations in middle-aged and older adults.

**Objective:** to investigate the association between alcohol consumption and physical limitations in Eastern European populations.

**Study design:** a cross-sectional survey of 28,783 randomly selected residents (45–69 years) in Novosibirsk (Russia), Krakow (Poland) and seven towns of Czech Republic.

**Methods:** physical limitations were defined as <75% of optimal physical functioning using the Physical Functioning (PF-10) Subscale of the Short-Form-36 questionnaire. Alcohol consumption was assessed by a graduated frequency questionnaire, and problem drinking was defined as ≥2 positive responses on the CAGE questionnaire. In the Russian sample, past drinking was also assessed.

**Results:** the odds of physical limitations were highest among non-drinkers, decreased with increasing drinking frequency, annual consumption and average drinking quantity and were not associated with problem drinking. The adjusted odds ratio (OR) of physical limitations in non-drinkers versus regular moderate drinkers was 1.61 (95% confidence interval: 1.48–1.75). In the Russian sample with past drinking available, the adjusted OR in those who stopped drinking for health reasons versus continuing drinkers was 3.19 (2.58–3.95); ORs in lifetime abstainers, former drinkers for non-health reasons and reduced drinkers for health reasons were 1.27 (1.02–1.57), 1.48 (1.18–1.85) and 2.40 (2.05–2.81), respectively.

**Conclusion:** this study found an inverse association between alcohol consumption and physical limitations. The high odds of physical limitations in non-drinkers can be largely explained by poor health of former drinkers. The apparently protective effect of heavier drinking was partly due to less healthy former heavy drinkers who moved to lower drinking categories.

## Background

Physical functioning, an essential component of health for older adults, is influenced by biological, lifestyle and environmental factors. Numerous studies have investigated the relationship between alcohol consumption and physical functioning [1–8]. A systematic review suggested that non-drinkers and heavy drinkers had a higher risk of functional limitations than light-to-moderate drinkers [9], although some studies reported no association [1] or increased risk only in non-drinkers [5, 7]. The protective effect of light-to-moderate drinking may be due to its cardio-protective and anti-inflammatory effects [4, 6].

The prevalence of physical limitations appears to be higher in Central and Eastern Europe (CEE) than in the west [10, 11]. Given the high alcohol consumption and high alcohol-attributable burden of disease in CEE [12, 13], it is plausible that alcohol may also play a role in the high rates of physical limitations. Older adults are more sensitive to alcohol than younger persons, because of their increased body fat and reduced body water that affect blood alcohol concentration, and negative interactions between alcohol and medications [14]. However, the evidence from CEE is sparse.

Besides potential harmful effects of heavy drinking, the literature suggests that abstainers have lower physical functioning [3–5, 7, 9]. Research on cardiovascular diseases (CVD) suggested that non-drinkers possibly include former drinkers who stopped drinking for health reasons (‘sick quitters’), which may spuriously overestimate the increased risk of CVD among non-drinkers [15, 16]. Similarly, some less healthy heavy drinkers move to light or non-drinking groups [15, 17]. This bias may equally affect studies of alcohol consumption and physical functioning. Despite the importance, we are not aware of any study directly addressing abstinence and/or reduced intake because of ill health in relation to physical limitations.

In this paper, we investigated the role of alcohol consumption on physical functioning in three ageing populations of CEE: whether heavy drinking is associated with lower functioning, and whether the apparently protective effect of moderate drinking (if found) can be explained by past drinking behaviour.

## Methods

### Study populations and subjects

We used data from the baseline survey (2002–2005) of Health, Alcohol and Psychosocial factors in Eastern Europe (HAPIEE) study in Novosibirsk (Russia), Krakow (Poland) and seven middle-sized towns in Czech Republic (Havirov/Karvina, Hradec Kralove, Jihlava, Kromeriz, Liberec and Usti nad Labem). Random samples of urban residents aged 45–69 years, stratified by sex and 5-year age groups, were selected from population registers (Czech towns and Krakow) and electoral lists (Novosibirsk) using a computerised procedure. A total of 28,783 participants were recruited. Participants completed a structured questionnaire either at home (Czech towns and Krakow) or in a clinic (Novosibirsk) and attended a short examination in a clinic [18]. The questionnaires were translated into local languages and back-translated into English to ensure cross-cultural comparability and piloted in a separate sample [19]. The study was approved by ethics committees at University College London and each local centre, and all participants signed informed consent.

### Measurements

Physical functioning was measured by the Physical Functioning Subscale (PF-10) of the Short-Form-36 (SF-36) questionnaire [20], which has been extensively validated in numerous countries, including those in this study (www.sf-36.com). The PF-10 assesses limitations on light, moderate and vigorous activities, mobility and self-care tasks. A score (0–100) was calculated, and the higher score indicates better physical functioning. The PF-10 was associated with objective physical performance in the expected direction (Supplementary data available in *Age and Ageing* online, Table S1). Participants with a score <75 were classified as having physical limitations.

Alcohol consumption in the last 12 months was evaluated by the graduated frequency (GF) questionnaire [21], with six levels of drinking quantity (≥10, 7–9, 5–6, 3–4, 1–2 and 0.5 drink during 1 day) and nine categories of drinking frequency (almost every day, 3–4/week, 1–2/week, 2–3/month, 1/month, 6–11/year, 3–5/year, 1–2/year and never). One standard drink was defined as 0.5 l of beer, 2 dl of wine or 50 ml of spirits, which roughly equals 20 g ethanol. Average drinking frequency, annual drinking amount and average drinking quantity per drinking day were derived, using the mid-points of drinking quantities and the corresponding frequencies. Average drinking quantity per day was categorised into non-drinkers, light, moderate and heavy drinkers (0, 0.1–19.9, 20.0–39.9, ≥40.0 g/day for women; 0, 0.1–39.9, 40.0–59.9, ≥60.0 g/day for men [22]); in line with previous research, the cut-offs for women were lower than for men [6, 8].

The drinking pattern combined drinking quantity and corresponding frequency in the GF. Light-to-moderate drinking was defined as ≤4 drinks during 1 day in men (≤2 drinks in women); higher intakes were considered as heavy drinking. Regular drinking was defined as ≥1/month; less than that was considered as irregular drinking. Problem drinking was classified as answering two or more positive responses to the CAGE questionnaire [23], in line with the previous study [4]. Both GF-based variables and problem drinking were strongly associated with separately taken measures of alcohol consumption and serum gamma-glutamyl transferase (Supplementary data available in *Age and Ageing* online, Table S2).

In Russia, past drinking behaviour was assessed by additional questions. Current non-drinkers were further categorised into lifetime abstainers and former drinkers. Similarly, current drinkers were also grouped into those who had reduced their consumption versus ‘continuing’ drinkers. Former drinkers and reduced drinkers were further categorised based on why they stopped/reduced drinking: health reasons versus other reasons.

### Covariates used in the analyses

Socioeconomic position (SEP) was evaluated by highest educational attainment, current economic activity and number of household amenities in childhood and adulthood, selected to be comparable across countries. Marital status was categorised into married/cohabiting, single, divorced and widowed. Body mass index (BMI) was calculated by objectively measured height and weight (kg/m^2^). Smoking status was classified into current, past and never smokers.

### Statistical methods

Among 28,783 eligible participants, 22,370 (77.7%) had complete information on all variables. Multiple imputations by chained equations (MICE) were used to handle missing data [24], and 20 imputed data sets were generated (Stata 12 commands available on request).

The association between alcohol consumption and physical limitations in the multiply imputed data sets was analysed by multivariate logistic regression. Two models were estimated: (i) adjusted for age and sex (and population, where appropriate) and (ii) additionally adjusted for SEP, marital status, BMI and smoking. All the data analyses were performed in Stata 12 using commands appropriate for imputed data (StataCorp, USA, 2011).

## Results

Table 1 presents the summary of study samples. Czechs had the lowest proportion of physical limitations while Poles had the highest. A larger proportion of Poles reported no drinking in the last year than their Czech and Russian counterparts. Czechs drank most frequently, while Russian men reported the highest consumption per drinking day and the highest prevalence of problem drinking.

**Table 1.** Descriptive characteristics of the study samples

	Country (*N*, %)		
	Czech Republic	Russia	Poland
Sex			
Men	4,070 (46.4)	4,239 (45.6)	5,219 (48.7)
Women	4,703 (53.6)	5,062 (54.4)	5,490 (51.3)
Age			
45.00–49.99	1,480 (16.9)	1,584 (17.0)	1,981 (18.5)
50.00–54.99	1,735 (19.8)	1,809 (19.5)	2,215 (20.7)
55.00–59.99	1,674 (19.1)	2,009 (21.6)	2,253 (21.0)
60.00–64.99	2,021 (23.0)	1,770 (19.0)	2,130 (19.9)
65.00–69.99	1,863 (21.2)	2,129 (22.9)	2,130 (19.9)
Physical functioning			
Good (≥75)	6,981 (79.6)	7,097 (76.3)	7,788 (72.7)
Poor (<75)	1,650 (18.8)	2,204 (23.7)	2,846 (26.6)
Missing	142 (1.6)	0	75 (0.7)
Average drinking frequency			
Never	1,090 (12.4)	1,472 (15.8)	3,673 (34.3)
<1/month	1,820 (20.8)	2,914 (31.3)	1,994 (18.6)
1–3/month	1,824 (20.8)	2,501 (26.9)	2,252 (21.0)
1–4/week	2,141 (24.4)	2,029 (21.8)	2,043 (19.1)
≥5/week	1,587 (18.1)	384 (4.1)	687 (6.4)
Missing	311 (3.5)	1 (<0.1)	60 (0.6)
Annual drinking volume (g ethanol)			
0	1,090 (12.4)	1,472 (15.8)	3,673 (34.3)
1–1,500^a^/1–250^b^	2,569 (29.3)	2,761 (29.7)	3,400 (31.8)
1,501–4,000^a^/251–500^b^	1,247 (14.2)	2,248 (24.2)	1,534 (14.3)
4,001–8,000^a^/501–1,500^b^	1,315 (15.0)	1,449 (15.6)	1,056 (9.9)
>8,000^a^/>1,500^b^	2,241 (25.5)	1,370 (14.7)	986 (9.2)
Missing	311 (3.5)	1 (<0.1)	60 (0.6)
Average drinking quantity per day			
Non-drinker	1,090 (12.4)	1,472 (15.8)	3,673 (34.3)
Light	4,153 (47.3)	1,978 (21.3)	4,673 (43.6)
Moderate	2,088 (23.8)	3,269 (35.2)	1,467 (13.7)
Heavy	1,131 (12.9)	2,581 (27.8)	836 (7.8)
Missing	311 (3.5)	1 (<0.1)	60 (0.6)
Drinking patterns			
Non-drinker	1,090 (12.4)	1,472 (15.8)	3,673 (34.3)
Irregular light-to-moderate	1,703 (19.4)	2,927 (31.5)	2,074 (19.4)
Regular light-to-moderate	2,643 (30.1)	2,012 (21.6)	2,857 (26.7)
Irregular heavy	1,884 (21.5)	1,312 (14.1)	1,413 (13.2)
Regular heavy	1,142 (13.0)	1,577 (17.0)	632 (5.9)
Missing	311 (3.5)	1 (<0.1)	60 (0.6)
Problem drinking			
No	7,870 (89.7)	8,415 (90.5)	8,014 (74.8)
Yes	453 (5.2)	885 (9.5)	509 (4.8)
Missing	450 (5.1)	1 (<0.1)	2,186 (20.4)
Total	8,773	9,301	10,709

^a^Among men.

^b^Among women.

Associations between alcohol consumption characteristics and physical limitations were similar across populations and sexes (Supplementary data available in *Age and Ageing* online, Tables S3–S5); therefore, the data were pooled. The results of pooled analyses are shown in Table 2. After adjustment for population, age and sex, the odds of physical limitations decreased with increasing drinking frequency, annual drinking amount, average drinking quantity per day and from non-hazardous to hazardous drinking pattern. The most notable was the increased odds in non-drinkers. This pattern persisted after additional adjustment for SEP, marital status, BMI and smoking. Problem drinking was not associated with physical limitations among drinkers.

**Table 2.** Odds ratios (95% confidence intervals) of physical limitations by alcohol consumption

	OR	
	Model 1^d^	Model 2^e^
Average drinking frequency		
0	2.03 (1.87, 2.21)	1.66 (1.52, 1.82)
<1/month	1.34 (1.24, 1.46)	1.25 (1.15, 1.37)
1–3/month	1.00	1.00
1–4/week	0.85 (0.77, 0.94)	0.89 (0.81, 0.99)
≥5/week	0.88 (0.77, 1.01)	0.94 (0.82, 1.08)
Annual drinking volume (g ethanol)		
0	1.56 (1.45, 1.68)	1.32 (1.22, 1.42)
1–1,500^a^/1–250^b^	1.00	1.00
1,501–4,000^a^/251–500^b^	0.77 (0.70, 0.84)	0.78 (0.71, 0.85)
4,001–8,000^a^/501–1,500^b^	0.62 (0.56, 0.69)	0.64 (0.58, 0.71)
>8,000^a^/>1,500^b^	0.69 (0.63, 0.77)	0.73 (0.66, 0.81)
Average drinking quantity per day		
Non-drinker	1.73 (1.60, 1.86)	1.39 (1.28, 1.50)
Light	1.00	1.00
Moderate	0.83 (0.76, 0.90)	0.80 (0.73, 0.87)
Heavy	0.88 (0.80, 0.97)	0.77 (0.69, 0.85)
Drinking pattern		
Non-drinker	2.05 (1.88, 2.22)	1.61 (1.48, 1.75)
Irregular light-to-moderate	1.35 (1.24, 1.46)	1.23 (1.12, 1.34)
Regular light-to-moderate	1.00	1.00
Irregular heavy	0.82 (0.74, 0.91)	0.78 (0.70, 0.87)
Regular heavy	0.94 (0.83, 1.05)	0.85 (0.75, 0.96)
Problem drinking^c^		
No	1.00	1.00
Yes	1.13 (0.99, 1.30)	1.05 (0.91, 1.21)

^a^Among men.

^b^Among women.

^c^Among drinkers.

^d^Adjusted for population, age and sex.

^e^Adjusted for population, age, sex, SEP (education, current economic activity, childhood amenities and adulthood amenities), marital status, BMI and smoking.

Table 3 shows the association of physical limitations with former and reduced drinking in the Russian sample. Compared with continuing drinkers, most participants who stopped/reduced alcohol intake had increased odds ratios (ORs) of physical limitations, but the ORs were highest among those who stopped/reduced drinking for health reasons. After further categorising continuing drinkers by drinking pattern, the associations between past drinking behaviour and physical limitations remained essentially the same (Supplementary data available in *Age and Ageing* online, Table S6).

**Table 3.** Odds ratios (95% confidence intervals) of physical limitations by abstinence or reduction of drinking in Russia

	OR	
	Model 1^a^	Model 2^b^
Men		
Lifetime abstainers	1.53 (0.70, 3.36)	1.39 (0.61, 3.14)
Former drinkers, health reasons	4.13 (3.01, 5.66)	2.86 (2.04, 4.03)
Former drinkers, non-health reasons	1.30 (0.90, 1.88)	1.21 (0.82, 1.77)
Reduced drinkers, health reasons	3.23 (2.56, 4.07)	2.57 (2.01, 3.30)
Reduced drinkers, non-health reasons	1.04 (0.82, 1.32)	0.91 (0.71, 1.18)
Continuing drinkers	1.00	1.00
Women		
Lifetime abstainers	1.44 (1.16, 1.79)	1.30 (1.04, 1.62)
Former drinkers, health reasons	4.03 (3.05, 5.31)	3.44 (2.59, 4.58)
Former drinkers, non-health reasons	1.90 (1.43, 2.52)	1.76 (1.31, 2.35)
Reduced drinkers, health reasons	2.27 (1.85, 2.78)	2.12 (1.72, 2.61)
Reduced drinkers, non-health reasons	0.91 (0.76, 1.09)	0.87 (0.72, 1.05)
Continuing drinkers	1.00	1.00
Both sexes		
Lifetime abstainers	1.45 (1.18, 1.79)	1.27 (1.02, 1.57)
Former drinkers, health reasons	4.01 (3.26, 4.93)	3.19 (2.58, 3.95)
Former drinkers, non-health reasons	1.60 (1.29, 1.99)	1.48 (1.18, 1.85)
Reduced drinkers, health reasons	2.67 (2.29, 3.10)	2.40 (2.05, 2.81)
Reduced drinkers, non-health reasons	0.95 (0.82, 1.10)	0.90 (0.77, 1.04)
Continuing drinkers	1.00	1.00

^a^Adjusted for age and sex (in analyses of both sexes).

^b^Adjusted for age, sex, SEP (education, current economic activity, childhood amenities and adulthood amenities), marital status, BMI and smoking.

## Discussion

In this large population-based study in CEE, we found that physical limitations were inversely associated with alcohol consumption, with the highest ORs among non-drinkers. However, much of the increased risk of physical limitations in non-drinkers was due to sick quitters. Similarly, some of the apparently protective effect of alcohol among drinkers appeared to be due to less healthy former heavy drinkers who reduced their intake and moved to lower drinking categories. There was no association between problem drinking and physical limitations.

Our study has several limitations. First, the cross-sectional nature of the data complicates the assessment of temporality, and we had to rely on self-reported past drinking behaviour. However, the history of alcohol-related health damage is likely to be under-reported due to social stigma, and one would expect under-reported stopping/reducing drinking for health reasons. In this case, the importance of past drinking behaviour in explaining the inverse association between alcohol consumption and physical limitations would also be under-estimated.

Second, the overall response rate was 60% [18], and we cannot eliminate a potential selection bias. It is likely that respondents differ from non-respondents in drinking behaviour, health status including physical limitations and other health behaviours. Middle-class subjects, who are more concerned about their health and less likely to drink heavily, are usually overrepresented in population surveys [25]; this may give rise to an overestimate of protective effect of moderate drinking. In addition, in western societies, long-term abstainers tend to be less healthy, less likely to be married and have lower SEP, less healthy lifestyle and worse social network than moderate drinkers [26, 27]. It is difficult to eliminate all potential residual confounding by these factors.

Third, participants may have under-reported their alcohol consumption and over-reported their physical functioning because of the social stigma attached to drinking and being unhealthy. However, physical limitations and alcohol consumption were associated with separately taken self-reports and objective makers, supporting the validity of the measurements. This bias is difficult to quantify; it would tend to under-estimate the role of past drinking behaviour and therefore not change the direction of our findings. Participants may have also over-reported their reduction of alcohol intake, and some of the ‘reduced drinkers’ are actually continuing drinkers. As in Russia the social stigma associated with alcohol affects women much more than men, the similarity of results in men and women points against the presence of a major bias.

Fourth, data on past drinking behaviour was only available in Russians. However, the pattern of associations between current drinking indices and physical limitations was very similar among the three populations, and it is reasonable to assume that if data on past drinking were available in Czechs and Poles, the results would be similar to those found in Novosibirsk.

Finally, we dealt with missing data by the MICE statistical technique to avoid loss of statistical power in complete-case analyses [28]. Additional complete-case analyses gave very similar results to those reported here, suggesting that missing data did not distort the results.

This study also has a number of strengths. It has large statistical power, collected extensive data on different aspects of alcohol consumption, adopted a widely used measure of physical functioning and examined rarely studied ageing populations in CEE. In particular, the availability of data on abstinence/reduction of drinking in studies in CEE is not common and, to our knowledge, valuable data on the reasons for stopping/reducing drinking have not been used in the region.

At the time these data were collected, the Czechs and Poles preferred beer and had similar per capita consumption (16.5 and 13.3 l, respectively) [29]; the Russian per capita consumption was similar (15.7 l), but Russia had the most risky drinking pattern with a preference of spirits [19, 29]. Despite these differences in drinking patterns, the associations between current drinking indices and physical limitations were similar across these three populations.

Given the relatively high levels of alcohol intake in these populations, we were surprised that the heaviest drinking groups and problem drinkers did not have increased odds of physical limitations. Instead, we found an inverse association between alcohol consumption and physical limitations, consistent with other studies [2–5, 7, 8], including a recent report from Russia, in which annual consumption of 10–19 l was associated with better physical health compared with non-drinking [30].

Most of previous studies, including the Russian report, did not consider former drinking. We are aware of only two studies, which examined former drinkers separately; one study found an increased risk of mobility limitations among male former drinkers [6]; the other reported no association between former drinking and functional limitations [8].

In our data, abstinence for health reasons was associated with an increased risk of physical limitations in both sexes, consistent with the hypothesis that non-drinkers include sick quitters [15]. However, abstinence for non-health reasons was associated with a relatively small increase in physical limitations, and only so among women. This gender difference may be related to misreporting; women may be more likely to report non-health reasons even when the true reasons were related to health.

With increasing age, decrease of alcohol consumption for health reasons might occur across all drinking groups [15]. In our data, drinkers who reduced drinking for health reasons indeed had higher odds of physical limitations than continuing drinkers, suggesting that their increased risk is due to their ill health, rather than low alcohol intake. Consistent with the results on stopping drinking, the reduction of drinking due to non-health reasons was not associated with physical limitations.

## Conclusion

We conclude that the inverse association between alcohol consumption and physical limitations in these ageing populations was partly explained by drinkers who reduced or quitted drinking due to health reasons. After excluding former drinkers, the results are consistent with a small (or no) protective effect for physical limitations. However, longitudinal studies that can directly address the issue of temporality are required to disentangle the effect of quitting or reducing drinking on physical functioning.

**Key points**

- Alcohol consumption was inversely associated with physical limitations in ageing populations in CEE.
- Non-drinkers had the highest odds of physical limitations, but no increased odds were found in the heaviest drinking group.
- The excess risk of physical limitations in non-drinkers was largely explained by poor health of former drinkers.
- The lower risk in heavy drinkers was partly due to former heavy drinkers who reduced drinking and moved to lower drinking groups.

## Supplementary data

Supplementary data mentioned in the text are available to subscribers in *Age and Ageing* online.

## Funding

The HAPIEE study was supported by the Wellcome Trust ‘Determinants of Cardiovascular Diseases in Eastern Europe: A Multi-centre Cohort Study’ (grant numbers 064947 and 081081), the US National Institute on Aging ‘Health Disparities and Aging in Societies in Transition (the HAPIEE study)’ (grant number 1R01 AG23522) and MacArthur Foundation Research Network ‘Health and Social Upheaval’ (grant number 712058).

## Conflicts of interest

None declared.

## Supplementary Material

Supplementary Data
