# Supplementary material for: Alcohol consumption and physical functioning among middle-aged and older adults in Central and Eastern Europe: Results from the HAPIEE study
Source: Age Ageing. 2014 Jun 30;44(1):84–9. doi: 10.1093/ageing/afu083 (PMC4255613; doi:10.1093/ageing/afu083)
Supplement: Supplementary Data [file supp_44_1_84__index.html]

Alcohol consumption and physical functioning among middle-aged and older adults in Central and Eastern Europe: Results from the HAPIEE study — Alcohol consumption and physical functioning among middle-aged and older adults in Central and Eastern Europe: Results from the HAPIEE study — Supplementary Data 

# Alcohol consumption and physical functioning among middle-aged and older adults in Central and Eastern Europe: Results from the HAPIEE study

## Supplementary Data

Supplementary Data

**Files in this Data Supplement:**

- Supplementary Data - Doc file
