# Supplementary material for: Alcohol consumption and physical functioning among middle-aged and older adults in Central and Eastern Europe: Results from the HAPIEE study
Source: Age Ageing. 2014 Jun 30;44(1):84–9. doi: 10.1093/ageing/afu083 (PMC4255613; doi:10.1093/ageing/afu083)
Supplement: Supplementary Data [file supp_afu083_afu083supp.doc]

Supplementary Data

Supplementary table 1. Baseline PF-10 score and objective physical performance at wave 2

| **Baseline PF-10 quartiles**^a^ | **Physical performance at wave 2 (2006-2008)** | | | | | |
| --- | --- | --- | --- | --- | --- | --- |
|  | **Grip strength** | | | **5 chair stands (seconds)** | | |
|  | Mean | SD | N | Mean | SD | N |
| **Czech Republic** |  |  |  |  |  |  |
| 1^st^ | 29.48 | 10.51 | 1403 | 11.82 | 4.28 | 1185 |
| 2^nd^ | 32.99 | 10.20 | 1463 | 10.23 | 3.81 | 1423 |
| 3^rd^ | 34.90 | 10.59 | 1415 | 9.32 | 3.67 | 1395 |
| 4^th^ | 36.45 | 10.94 | 864 | 8.68 | 3.30 | 851 |
| **Russia** |  |  |  |  |  |  |
| 1^st^ | 28.31 | 9.45 | 1640 | 12.90 | 3.86 | 1358 |
| 2^nd^ | 32.44 | 10.9 | 1935 | 11.55 | 3.33 | 1796 |
| 3^rd^ | 37.32 | 10.55 | 1233 | 10.71 | 3.07 | 1194 |
| 4^th^ | 39.66 | 10.93 | 1282 | 10.22 | 2.89 | 1253 |
| **Poland** |  |  |  |  |  |  |
| 1^st^ | 27.95 | 10.34 | 1614 | 12.58 | 4.77 | 1370 |
| 2^nd^ | 31.66 | 10.23 | 2306 | 10.92 | 3.74 | 2205 |
| 3^rd^ | 35.19 | 10.62 | 1077 | 10.03 | 3.33 | 1046 |
| 4^th^ | 36.47 | 10.73 | 1432 | 9.75 | 3.24 | 1392 |

Note: ^a^ Country-specific baseline PF-10 quartiles

Supplementary table 2. GF-based alcohol indices, problem drinking and other alcohol measures at baseline

|  | **Men** | | | | | | | | | | **Women** | | | | | | | | | |
| --- | --- | --- | --- | --- | --- | --- | --- | --- | --- | --- | --- | --- | --- | --- | --- | --- | --- | --- | --- | --- |
|  | **Weekly alcohol intake (g/day)^a^** | | **Alcohol intake from FFQ (g/day)^b^** | | **GGT (Russia)^c^** | | | **GGT (Czech Republic & Poland)^c^** | | | **Weekly alcohol intake (g/day)^a^** | | **Alcohol intake from FFQ (g/day)^b^** | | **GGT (Russia)^c^** | | | **GGT (Czech Republic & Poland)^c^** | | |
|  | Median | N | Median | N | Mean | SD | N | Mean | SD | N | Median | N | Median | N | Mean | SD | N | Mean | SD | N |
| **Average drinking frequency** |  |  |  |  |  |  |  |  |  |  |  |  |  |  |  |  |  |  |  |  |
| Never | 0 | 1958 | 0 | 1793 | 30.07 | 35.02 | 569 | 31.64 | 65.31 | 471 | 0 | 4227 | 0 | 3833 | 27.32 | 21.31 | 892 | 24.40 | 54.84 | 532 |
| <1/month | 0 | 1863 | 0.65 | 1703 | 32.34 | 27.22 | 585 | 28.79 | 48.54 | 452 | 0 | 4749 | 0.59 | 4514 | 29.29 | 30.42 | 2313 | 23.24 | 44.49 | 377 |
| 1-3/month | 1.19 | 2956 | 2.05 | 2768 | 37.49 | 47.36 | 1086 | 35.31 | 57.33 | 597 | 0 | 3537 | 1.24 | 3388 | 28.90 | 23.29 | 1407 | 18.90 | 42.88 | 308 |
| 1-4/week | 12.86 | 4307 | 6.84 | 3986 | 43.08 | 46.98 | 1621 | 42.32 | 74.83 | 809 | 5.71 | 1876 | 3.78 | 1755 | 33.01 | 32.91 | 397 | 34.44 | 91.38 | 203 |
| ≥5/week | 36.00 | 2209 | 19.00 | 2030 | 55.13 | 83.58 | 359 | 57.98 | 100.23 | 619 | 17.14 | 441 | 10.00 | 410 | 33.00 | 22.83 | 24 | 64.90 | 156.97 | 49 |
| **Annual drinking volume (g)** |  |  |  |  |  |  |  |  |  |  |  |  |  |  |  |  |  |  |  |  |
| 0 | 0 | 1958 | 0 | 1793 | 30.07 | 35.02 | 569 | 31.64 | 65.31 | 471 | 0 | 4227 | 0 | 3833 | 27.32 | 21.31 | 892 | 24.40 | 54.84 | 532 |
| 1-1500 | 0 | 4430 | 1.30 | 4065 | 33.02 | 31.88 | 1188 | 31.95 | 57.07 | 1099 | 0 | 4146 | 0 | 3921 | 28.13 | 25.48 | 1559 | 22.62 | 47.92 | 419 |
| 1501-4000 | 7.43 | 2483 | 4.95 | 2320 | 39.94 | 52.05 | 820 | 42.51 | 75.83 | 498 | 0 | 2506 | 0.65 | 2412 | 29.21 | 30.71 | 1417 | 18.83 | 33.51 | 161 |
| 4001-8000 | 15.43 | 1767 | 8.60 | 1648 | 42.55 | 46.19 | 683 | 40.33 | 67.51 | 335 | 1.14 | 2032 | 1.40 | 1947 | 30.46 | 26.10 | 759 | 18.83 | 35.67 | 167 |
| >8000 | 34.29 | 2655 | 17.60 | 2454 | 50.21 | 64.89 | 960 | 63.17 | 102.40 | 545 | 8.57 | 1919 | 4.52 | 1787 | 34.32 | 34.13 | 406 | 47.90 | 122.19 | 190 |
| **Average drinking quantity per day** |  |  |  |  |  |  |  |  |  |  |  |  |  |  |  |  |  |  |  |  |
| Non-drinker | 0 | 1958 | 0 | 1793 | 30.07 | 35.02 | 569 | 31.64 | 65.31 | 471 | 0 | 4227 | 0 | 3833 | 27.32 | 21.31 | 892 | 24.40 | 54.84 | 532 |
| Light | 9.43 | 6630 | 4.30 | 6061 | 36.46 | 42.84 | 1011 | 39.07 | 74.41 | 1864 | 0 | 4021 | 0.65 | 3782 | 28.15 | 22.85 | 958 | 23.19 | 58.10 | 455 |
| Moderate | 11.43 | 1497 | 6.36 | 1426 | 40.77 | 53.47 | 765 | 47.00 | 59.23 | 209 | 0 | 5266 | 0.65 | 5036 | 28.86 | 26.12 | 2486 | 26.63 | 67.96 | 402 |
| Heavy | 11.43 | 3208 | 7.02 | 3000 | 43.31 | 51.70 | 1875 | 53.41 | 84.82 | 404 | 0 | 1316 | 1.26 | 1249 | 33.86 | 40.42 | 697 | 43.72 | 105.04 | 80 |
| **Drinking pattern** |  |  |  |  |  |  |  |  |  |  |  |  |  |  |  |  |  |  |  |  |
| Non-drinker | 0 | 1958 | 0 | 1793 | 30.07 | 35.02 | 569 | 31.64 | 65.31 | 471 | 0 | 4227 | 0 | 3833 | 27.32 | 21.31 | 892 | 24.40 | 54.84 | 532 |
| Irregular light-to-moderate | 0 | 1801 | 0.65 | 1660 | 31.37 | 25.63 | 546 | 28.87 | 50.33 | 452 | 0 | 4788 | 0.59 | 4567 | 28.57 | 25.96 | 2366 | 22.96 | 42.71 | 384 |
| Regular light-to-moderate | 10.29 | 4516 | 4.95 | 4139 | 40.23 | 50.60 | 1195 | 40.79 | 71.53 | 1150 | 2.86 | 2928 | 1.40 | 2769 | 28.29 | 23.09 | 806 | 23.40 | 63.37 | 311 |
| Irregular heavy | 11.43 | 2572 | 5.54 | 2409 | 36.48 | 27.97 | 645 | 40.15 | 76.38 | 552 | 0 | 1995 | 1.40 | 1902 | 31.97 | 37.90 | 660 | 25.27 | 67.86 | 161 |
| Regular heavy | 25.71 | 2446 | 10.40 | 2279 | 47.84 | 63.12 | 1265 | 68.44 | 103.89 | 323 | 7.14 | 892 | 3.31 | 829 | 34.95 | 34.32 | 309 | 56.73 | 137.95 | 81 |
| **Problem drinking** |  |  |  |  |  |  |  |  |  |  |  |  |  |  |  |  |  |  |  |  |
| No | 5.71 | 10977 | 3.87 | 10156 | 36.69 | 39.75 | 3409 | 37.04 | 65.71 | 2503 | 0 | 13078 | 0.65 | 12365 | 28.89 | 26.52 | 4963 | 25.86 | 63.30 | 1230 |
| Yes | 25.14 | 1622 | 10.59 | 1515 | 50.93 | 72.98 | 811 | 84.72 | 134.23 | 230 | 12.46 | 213 | 5.54 | 197 | 46.96 | 59.56 | 70 | 90.65 | 190.72 | 20 |

Note: ^a^ Separate questions on beverage-specific alcohol intake during one week; ^b^ FFQ: food frequency questionnaire (separate questionnaire); ^c^ GGT: gamma-glutamyl transferase; please note that the GGT in Russia was analysed in a different laboratory from the GGT in Czech Republic and Poland.

Supplementary table 3. Odds ratios (95% confidence intervals) of physical limitations by alcohol consumption, Czech Republic

|  | **Men** | | **Women** | |
| --- | --- | --- | --- | --- |
|  | Model 1^1^ | Model 2^2^ | Model 1^1^ | Model 2^2^ |
| **Average drinking frequency** |  |  |  |  |
| 0 | 2.72 (1.96, 3.78) | 1.80 (1.24, 2.60) | 2.37 (1.91, 2.95) | 1.62 (1.27, 2.05) |
| <1/month | 1.22 (0.91, 1.64) | 1.26 (0.91, 1.74) | 1.38 (1.12, 1.69) | 1.23 (0.98, 1.53) |
| 1-3/month | 1.00 | 1.00 | 1.00 | 1.00 |
| 1-4/week^a^ | 0.83 (0.64, 1.09) | 0.90 (0.67, 1.20) | -- | -- |
| ≥5/week^a^ | 0.71 (0.54, 0.93) | 0.75 (0.56, 1.00) | -- | -- |
| ≥1/week^b^ | -- | -- | 0.88 (0.70, 1.10) | 0.94 (0.74, 1.20) |
| **Annual drinking volume (g ethanol)** |  |  |  |  |
| 0 | 2.52 (1.87, 3.39) | 1.60 (1.15, 2.23) | 1.79 (1.48, 2.17) | 1.35 (1.09, 1.66) |
| 1-1500^a^ /1-250^b^ | 1.00 | 1.00 | 1.00 | 1.00 |
| 1501-4000^a^ /251-500^b^ | 0.82 (0.63, 1.06) | 0.86 (0.65, 1.14) | 0.90 (0.70, 1.16) | 0.95 (0.73, 1.24) |
| 4001-8000^a^ /501-1500^b^ | 0.66 (0.49, 0.89) | 0.69 (0.50, 0.96) | 0.67 (0.52, 0.86) | 0.77 (0.59, 1.00) |
| >8000^a^ />1500^b^ | 0.76 (0.60, 0.95) | 0.73 (0.57, 0.94) | 0.71 (0.57, 0.88) | 0.81 (0.64, 1.02) |
| **Average drinking quantity per day** |  |  |  |  |
| Non-drinker | 3.11 (2.35, 4.11) | 1.89 (1.38, 2.59) | 2.05 (1.69, 2.49) | 1.41 (1.14, 1.74) |
| Light | 1.00 | 1.00 | 1.00 | 1.00 |
| Moderate | 0.88 (0.63, 1.24) | 0.78 (0.54, 1.13) | 0.88 (0.73, 1.05) | 0.86 (0.71, 1.04) |
| Heavy | 1.22 (0.97, 1.54) | 1.02 (0.79, 1.32) | 1.08 (0.82, 1.43) | 0.99 (0.73, 1.34) |
| **Drinking pattern** |  |  |  |  |
| Non-drinker | 2.96 (2.20, 3.98) | 1.89 (1.35, 2.64) | 2.26 (1.84, 2.78) | 1.55 (1.23, 1.94) |
| Irregular light-to-moderate | 1.39 (1.08, 1.80) | 1.40 (1.06, 1.85) | 1.27 (1.03, 1.55) | 1.14 (0.92, 1.42) |
| Regular light-to-moderate | 1.00 | 1.00 | 1.00 | 1.00 |
| Irregular heavy | 0.73 (0.56, 0.93) | 0.77 (0.59, 1.01) | 0.74 (0.57, 0.95) | 0.81 (0.62, 1.05) |
| Regular heavy | 0.99 (0.76, 1.30) | 0.95 (0.71, 1.27) | 0.97 (0.72, 1.32) | 0.96 (0.69, 1.33) |
| **Problem drinking**^c^ |  |  |  |  |
| No | 1.00 | 1.00 | -- | -- |
| Yes | 1.26 (0.93, 1.71) | 1.12 (0.80, 1.58) | -- | -- |

^a^ Among men, ^b^ Among women, ^c^ Among drinkers

^1^ Adjusted for age

^2^ Adjusted for age, SEP (education, current economic activity, childhood amenities and adulthood amenities), marital status, BMI and smoking

Supplementary table 4. Odds ratios (95% confidence intervals) of physical limitations by alcohol consumption, Russia

|  | **Men** | | **Women** | |
| --- | --- | --- | --- | --- |
|  | Model 1^1^ | Model 2^2^ | Model 1^1^ | Model 2^2^ |
| **Average drinking frequency** |  |  |  |  |
| 0 | 1.69 (1.30, 2.20) | 1.61 (1.21, 2.13) | 1.95 (1.62, 2.34) | 1.71 (1.41, 2.07) |
| <1/month | 1.29 (0.99, 1.68) | 1.34 (1.01, 1.79) | 1.09 (0.94, 1.28) | 1.02 (0.87, 1.19) |
| 1-3/month | 1.00 | 1.00 | 1.00 | 1.00 |
| 1-4/week^a^ | 0.86 (0.69, 1.08) | 1.01 (0.79, 1.28) | -- | -- |
| ≥5/week^a^ | 0.80 (0.55, 1.17) | 0.92 (0.62, 1.36) | -- | -- |
| ≥1/week^b^ | -- | -- | 0.83 (0.63, 1.08) | 0.86 (0.65, 1.13) |
| **Annual drinking volume (g ethanol)** |  |  |  |  |
| 0 | 1.43 (1.11, 1.84) | 1.25 (0.95, 1.63) | 1.53 (1.29, 1.81) | 1.39 (1.17, 1.66) |
| 1-1500^a^ /1-250^b^ | 1.00 | 1.00 | 1.00 | 1.00 |
| 1501-4000^a^ /251-500^b^ | 0.85 (0.66, 1.08) | 0.78 (0.60, 1.01) | 0.70 (0.59, 0.82) | 0.70 (0.59, 0.82) |
| 4001-8000^a^ /501-1500^b^ | 0.64 (0.48, 0.86) | 0.66 (0.49, 0.90) | 0.64 (0.52, 0.78) | 0.62 (0.50, 0.76) |
| >8000^a^ />1500^b^ | 0.69 (0.53, 0.89) | 0.71 (0.54, 0.94) | 0.83 (0.64, 1.07) | 0.84 (0.64, 1.10) |
| **Average drinking quantity per day** |  |  |  |  |
| Non-drinker | 1.30 (1.00, 1.68) | 1.05 (0.79, 1.38) | 1.75 (1.44, 2.12) | 1.53 (1.25, 1.87) |
| Light | 1.00 | 1.00 | 1.00 | 1.00 |
| Moderate | 0.54 (0.41, 0.72) | 0.57 (0.42, 0.77) | 0.93 (0.79, 1.10) | 0.89 (0.75, 1.06) |
| Heavy | 0.69 (0.56, 0.86) | 0.58 (0.46, 0.73) | 0.84 (0.67, 1.05) | 0.73 (0.58, 0.93) |
| **Drinking pattern** |  |  |  |  |
| Non-drinker | 1.55 (1.20, 2.01) | 1.28 (0.97, 1.69) | 1.97 (1.59, 2.43) | 1.65 (1.32, 2.05) |
| Irregular light-to-moderate | 1.12 (0.86, 1.46) | 1.09 (0.81, 1.45) | 1.13 (0.94, 1.36) | 1.03 (0.85, 1.25) |
| Regular light-to-moderate | 1.00 | 1.00 | 1.00 | 1.00 |
| Irregular heavy | 0.73 (0.55, 0.97) | 0.70 (0.51, 0.95) | 0.73 (0.57, 0.94) | 0.61 (0.47, 0.79) |
| Regular heavy | 0.77 (0.61, 0.97) | 0.67 (0.52, 0.86) | 1.25 (0.92, 1.69) | 1.15 (0.84, 1.57) |
| **Problem drinking**^c^ |  |  |  |  |
| No | 1.00 | 1.00 | -- | -- |
| Yes | 0.94 (0.74, 1.20) | 0.82 (0.63, 1.05) | -- | -- |

^a^ Among men, ^b^ Among women, ^c^ Among drinkers

^1^ Adjusted for age

^2^ Adjusted for age, SEP (education, current economic activity, childhood amenities and adulthood amenities), marital status, BMI and smoking

Supplementary table 5. Odds ratios (95% confidence intervals) of physical limitations by alcohol consumption, Poland

|  | **Men** | | **Women** | |
| --- | --- | --- | --- | --- |
|  | Model 1^1^ | Model 2^2^ | Model 1^1^ | Model 2^2^ |
| **Average drinking frequency** |  |  |  |  |
| 0 | 1.94 (1.59, 2.37) | 1.63 (1.32, 2.01) | 2.26 (1.90, 2.69) | 1.91 (1.59, 2.30) |
| <1/month | 1.55 (1.24, 1.95) | 1.52 (1.19, 1.93) | 1.66 (1.37, 2.02) | 1.54 (1.26, 1.89) |
| 1-3/month | 1.00 | 1.00 | 1.00 | 1.00 |
| 1-4/week^a^ | 0.98 (0.79, 1.20) | 0.99 (0.80, 1.23) | -- | -- |
| ≥5/week^a^ | 0.89 (0.68, 1.18) | 0.89 (0.66, 1.19) | -- | -- |
| ≥1/week^b^ | -- | -- | 0.94 (0.74, 1.21) | 1.03 (0.80, 1.33) |
| **Annual drinking volume (g ethanol)** |  |  |  |  |
| 0 | 1.54 (1.30, 1.82) | 1.27 (1.06, 1.52) | 1.44 (1.25, 1.67) | 1.29 (1.11, 1.50) |
| 1-1500^a^ /1-250^b^ | 1.00 | 1.00 | 1.00 | 1.00 |
| 1501-4000^a^ /251-500^b^ | 0.73 (0.59, 0.90) | 0.73 (0.58, 0.91) | 0.71 (0.56, 0.90) | 0.75 (0.59, 0.95) |
| 4001-8000^a^ /501-1500^b^ | 0.61 (0.45, 0.81) | 0.59 (0.43, 0.79) | 0.57 (0.45, 0.73) | 0.60 (0.47, 0.77) |
| >8000^a^ />1500^b^ | 0.75 (0.58, 0.98) | 0.68 (0.51, 0.90) | 0.63 (0.48, 0.82) | 0.73 (0.55, 0.97) |
| **Average drinking quantity per day** |  |  |  |  |
| Non-drinker | 1.74 (1.49, 2.05) | 1.41 (1.19, 1.67) | 1.60 (1.40, 1.84) | 1.33 (1.15, 1.54) |
| Light | 1.00 | 1.00 | 1.00 | 1.00 |
| Moderate | 0.79 (0.58, 1.09) | 0.73 (0.52, 1.02) | 0.72 (0.60, 0.87) | 0.69 (0.57, 0.84) |
| Heavy | 0.96 (0.76, 1.21) | 0.81 (0.63, 1.03) | 0.98 (0.69, 1.39) | 0.80 (0.55, 1.15) |
| **Drinking pattern** |  |  |  |  |
| Non-drinker | 1.94 (1.63, 2.33) | 1.55 (1.28, 1.87) | 2.35 (1.97, 2.80) | 1.90 (1.58, 2.29) |
| Irregular light-to-moderate | 1.69 (1.38, 2.07) | 1.60 (1.29, 1.99) | 1.60 (1.31, 1.95) | 1.44 (1.18, 1.77) |
| Regular light-to-moderate | 1.00 | 1.00 | 1.00 | 1.00 |
| Irregular heavy | 0.81 (0.64, 1.02) | 0.73 (0.58, 0.93) | 1.36 (1.04, 1.77) | 1.29 (0.97, 1.70) |
| Regular heavy | 0.98 (0.74, 1.31) | 0.79 (0.58, 1.06) | 0.85 (0.54, 1.36) | 0.81 (0.50, 1.30) |
| **Problem drinking**^c^ |  |  |  |  |
| No | 1.00 | 1.00 | -- | -- |
| Yes | 1.09 (0.83, 1.43) | 0.95 (0.72, 1.27) | -- | -- |

^a^ Among men, ^b^ Among women, ^c^ Among drinkers

^1^ Adjusted for age

^2^ Adjusted for age, SEP (education, current economic activity, childhood amenities and adulthood amenities), marital status, BMI and smoking

Supplementary table 6. Odds ratios (95% confidence intervals) of physical limitations by abstinence/reduction of drinking and drinking pattern in Russia

|  | OR | |
| --- | --- | --- |
|  | Model 1^1^ | Model 2^2^ |
|  |  |  |
| **Men** |  |  |
| Lifetime abstainers | 1.44 (0.64, 3.24) | 1.22 (0.53, 2.83) |
| Former drinkers, health reasons | 3.88 (2.68, 5.61) | 2.48 (1.67, 3.70) |
| Former drinkers, non-health reasons | 1.22 (0.81, 1.85) | 1.04 (0.67, 1.61) |
| Reduced drinkers, health reasons | 3.04 (2.25, 4.10) | 2.23 (1.62, 3.08) |
| Reduced drinkers, non-health reasons | 0.98 (0.72, 1.33) | 0.79 (0.57, 1.10) |
| Irregular light-to-moderate | 1.26 (0.83, 1.92) | 1.32 (0.85, 2.07) |
| Regular light-to-moderate | 1.00 | 1.00 |
| Irregular heavy | 0.67 (0.43, 1.07) | 0.64 (0.40, 1.04) |
| Regular heavy | 0.89 (0.64, 1.25) | 0.73 (0.51, 1.04) |
|  |  |  |
| **Women** |  |  |
| Lifetime abstainers | 1.50 (1.12, 2.00) | 1.22 (0.91, 1.65) |
| Former drinkers, health reasons | 4.18 (3.00, 5.84) | 3.25 (2.30, 4.58) |
| Former drinkers, non-health reasons | 1.97 (1.40, 2.77) | 1.66 (1.17, 2.35) |
| Reduced drinkers, health reasons | 2.35 (1.79, 3.10) | 1.99 (1.50, 2.64) |
| Reduced drinkers, non-health reasons | 0.94 (0.73, 1.22) | 0.82 (0.63, 1.07) |
| Irregular light-to-moderate | 1.13 (0.90, 1.43) | 1.01 (0.80, 1.29) |
| Regular light-to-moderate | 1.00 | 1.00 |
| Irregular heavy | 0.69 (0.50, 0.94) | 0.58 (0.42, 0.81) |
| Regular heavy | 1.25 (0.87, 1.80) | 1.14 (0.78, 1.65) |
|  |  |  |
| **Both sexes** |  |  |
| Lifetime abstainers | 1.47 (1.15, 1.90) | 1.16 (0.89, 1.51) |
| Former drinkers, health reasons | 4.01 (3.14, 5.14) | 2.87 (2.22, 3.71) |
| Former drinkers, non-health reasons | 1.60 (1.24, 2.07) | 1.33 (1.02, 1.73) |
| Reduced drinkers, health reasons | 2.67 (2.18, 3.27) | 2.15 (1.74, 2.65) |
| Reduced drinkers, non-health reasons | 0.95 (0.78, 1.16) | 0.81 (0.66, 0.99) |
| Irregular light-to-moderate | 1.13 (0.94, 1.37) | 1.01 (0.83, 1.23) |
| Regular light-to-moderate | 1.00 | 1.00 |
| Irregular heavy | 0.68 (0.53, 0.88) | 0.58 (0.44, 0.76) |
| Regular heavy | 1.01 (0.79, 1.28) | 0.85 (0.66, 1.09) |
|  |  |  |

^1^ Adjusted for age and sex (in analyses of both sexes)

^2^ Adjusted for age, sex, SEP (education, current economic activity, childhood amenities and adulthood amenities), marital status, BMI and smoking
